# P-1980. Decoding the Complexity of Post COVID Conditions: Transcriptomic Analyses of Machine-Learning-Based Chronic Symptom Phenotypes

**DOI:** 10.1093/ofid/ofae631.2138

**Published:** 2025-01-29

**Authors:** Nusrat J Epsi, Josh Chenoweth, Stephanie A Richard, David A Lindholm, Katrin Mende, Anuradha Ganesan, Tahaniyat Lalani, Rhonda E Colombo, Derek Larson, Catherine Berjohn, David Saunders, Mark P Simons, Robert O’Connell, David R Tribble, Brian Agan, Timothy Burgess, Clifton Dalgard, Simon Pollett

**Affiliations:** IDCRP HJF, Bethesda, Maryland; Henry M. Jackson Foundation, Bethesda, Maryland; Infectious Disease Clinical Research Program, Department of Preventive Medicine and Biostatistics, Uniformed Services University of the Health Sciences, Bethesda, MD, USA, Bethesda, Maryland; Department of Medicine, Uniformed Services University of the Health Sciences; Brooke Army Medical Center, San Antonio, TX; Infectious Disease Clincial Research Program, JBSA Ft Sam Houston, Texas; Infectious Disease Clinical Research Program, USUHS; Henry M. Jackson Foundation for the Advancement of Military Medicine Inc, Bethesda, Maryland; Naval Medical Center Portsmouth, Portsmouth, Virginia; Infectious Disease Clinical Research Program, USUHS; Henry M. Jackson Foundation for the Advancement of Military Medicine, Inc., Bethesda, Maryland; Fort Belvoir Community Hospital and Uniformed Services University, Fort Belvoir, Virginia; Naval Medical Center San Diego, San Diego, California; Uniformed Services University of the Health Sciences, Bethesda, MD, USA, Bethesda, Maryland; Infectious Disease Clinical Research Program, Department of Preventive Medicine and Biostatistics, Uniformed Services University of the Health Sciences, Bethesda, MD, USA, Bethesda, Maryland; Infectious Disease Clinical Research Program, USUHS, Bethesda, Maryland; Uniformed Services University of the Health Sciences, Bethesda, Maryland; Infectious Disease Clinical Research Program, Department of Preventive Medicine and Biostatistics, Uniformed Services University of the Health Sciences, Bethesda, MD, USA, Bethesda, Maryland; Infectious Disease Clinical Research Program, Department of Preventive Medicine and Biostatistics, Uniformed Services University of the Health Sciences, Bethesda, MD, USA, Bethesda, Maryland; The American Genome Center, Uniformed Services University of the Health Sciences, Bethesda, Maryland; Infectious Disease Clinical Research Program, Department of Preventive Medicine and Biostatistics, Uniformed Services University of the Health Sciences, Bethesda, MD, USA, Bethesda, Maryland

## Abstract

**Background:**

Post-COVID conditions (PCC, ‘Long COVID’) remain a military health concern, and symptom heterogeneity limits understanding of PCC pathogenesis. To address this, we used machine learning to define PCC phenotypes in Military Health System (MHS) beneficiaries. Here, we extend these findings to identify early transcriptomic profiles which may predict the development of these distinct symptom-based PCC phenotypes.

**Figure:**
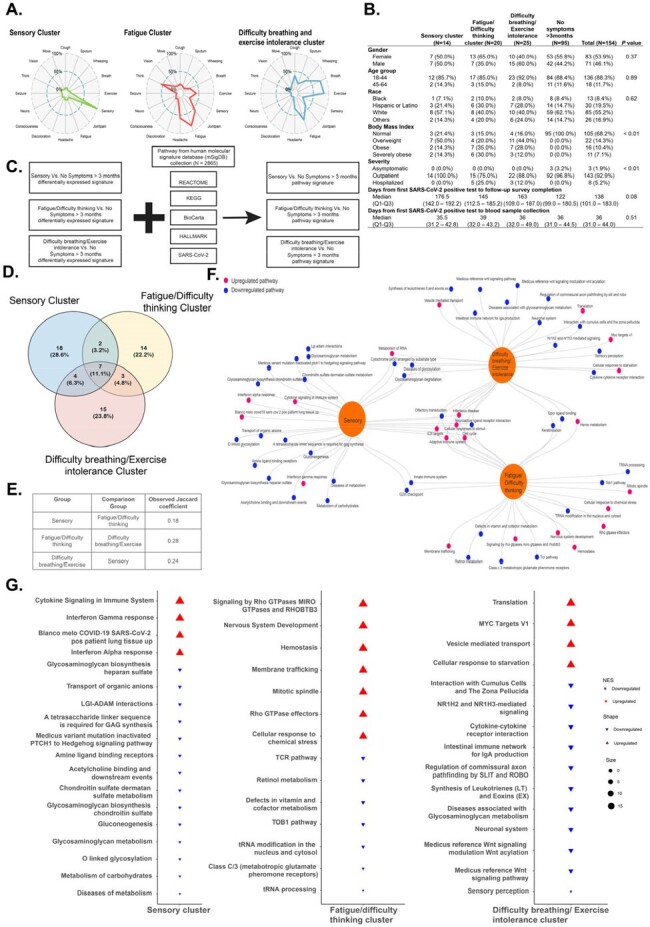

(A) Machine learning techniques identified three groups of participants based on chronic symptom-clustering. Radial charts display the distribution of post-COVID condition (PCC) symptoms. Each plot’s axes represent different symptoms, with the length of each spoke indicating symptom prevalence. Shorter spokes indicate lower prevalence, while longer spokes indicate higher prevalence. (B) Clinical and demographic characteristics of 154 military health system beneficiaries by identified symptom-clusters. (C) Schematic representation of pathway enrichment analysis using the differentially expressed signature and pathway databases. (D-E) Jaccard similarity analysis identifies the similarity of leading-edge pathways within each symptom-cluster phenotype, with a result close to 1 indicating high similarity and close to 0 indicating significant differences. (F) Network analysis depicts pathway interactions between each symptom-cluster phenotype, with larger nodes representing clusters and smaller nodes indicating individual pathways. Grey lines indicate associations between clusters and pathways, revealing both overlapping and distinct pathway sets. Upregulated and downregulated pathways are highlighted in red and blue, respectively. (G) Non-overlapping leading-edge pathways are identified, with the size of each pathway node corresponding to the normalized enrichment score (NES), with an upward arrow and red color indicates upregulation, while a downward arrow and blue color denotes downregulation.

**Methods:**

The EPICC cohort study evaluates SARS-CoV-2 infection in MHS beneficiaries. PCC phenotypes were identified using symptom data; transcriptome profiling was performed on early post-infection blood samples from participants who did and did not develop 3-month symptom PCC phenotypes. Participants with pre-COVID comorbidities were excluded to delineate gene responses for PCC from pre-existing illnesses. Comparison of transcriptomic signatures associated with symptom clusters involved utilizing Reactome, KEGG, BioCarta, and Hallmark pathway enrichment analysis. Hypergeometric *p*-values and the Jaccard coefficient (JC) assess pathway distinctiveness between symptom clusters and those with no PCC.

**Results:**

We analyzed 154 participants with no reported comorbidities (Fig. 1), who completed a symptom survey 3-months after a SARS-CoV-2 positive test, and who had an early blood sample (median: 36 days post-infection). Clustering analysis stratified 3 distinct PCC symptom clusters: Sensory, Fatigue/difficulty thinking, and Difficulty breathing/exercise intolerance. Transcriptomic analysis noted post-infection signatures which predicted the development of each cluster (JC: 0.18–0.28). Leading-edge pathways included cytokine signaling in the Sensory cluster (Normalized Enrichment Score, NES=16.16, *P* < 0.001), nervous system development in the Fatigue/difficulty thinking cluster (NES=10.23, *P* < 0.001), and MYC Targets V1 in the Difficulty thinking/exercise intolerance cluster (NES=8.89, *P* < 0.001).

**Conclusion:**

Distinct symptom-based PCC phenotypes are associated with different early post-infection gene expression pathways. These findings inform mechanistic hypotheses and the development of PCC prediction tools. These methods may be applied for prediction of post-acute sequelae in other pathogens.

**Disclosures:**

Timothy Burgess, MD, MPH, AstraZeneca: The IDCRP and HJF were funded to conduct an unrelated phase III COVID-19 monoclonal antibody immunoprophylaxis trial as part of US Govt COVID Response Simon Pollett, MBBS, AstraZeneca: The IDCRP and HJF were funded to conduct an unrelated phase III COVID-19 monoclonal antibody immunoprophylaxis trial as part of US Govt COVID Response

